# THE ASSOCIATION OF PERIPHERAL AND CENTRAL OLFACTION WITH FRAILTY IN OLDER ADULTS

**DOI:** 10.1093/geroni/igae098.0655

**Published:** 2024-12-31

**Authors:** Nicholas Rowan, Nimesh Nagururu, Isaac Bernstein, Kristin Voegtline, Sarah Olson, Yuri Agrawal

**Affiliations:** Johns Hopkins University School of Medicine, Baltimore, Maryland, United States; Johns Hopkins University School of Medicine, Baltimore, Maryland, United States; Johns Hopkins University School of Medicine, Baltimore, Maryland, United States; Johns Hopkins University School of Medicine, Department of Pediatrics, Baltimore, Maryland, United States; Johns Hopkins University, Baltimore, Maryland, United States; Johns Hopkins University School of Medicine: Department of Otolaryngology-Head and Neck Surgery, Baltimore, Maryland, United States

## Abstract

Olfactory impairment is increasingly recognized as a biomarker of frailty, but the relationship between olfactory subdomains that describe peripheral or central dysfunction and frailty remains unexplored. In this study, we examined 1,160 older adults from the National Social Life, Health, and Aging Project Wave 3. Olfactory identification (OI): the ability to identify an odorant; and olfactory sensitivity (OS): the ability to detect the presence of an odorant, were assessed using 5 and 6-point measures respectively. Frailty was operationalized as both a 37-item Frailty Index (FI) and the 5-item Physical Frailty Phenotype (PFP). Mixed models were fit to examine the association between OI, OS, FI and PFP, while adjusting for demographic and clinical covariates. Subjects in the most-frail PFP category had lower OI and OS scores (OI: 3.88 vs 4.19, p= 0.016; OS: 3.15 vs 3.47, p= 0.031), whereas, subjects in the most-frail FI category exhibited lower OI scores but not OS scores when compared to non-frail subjects (OI: 3.72 vs 4.27, p= 0.014; OS: 3.19 vs 3.43, p= 0.476). Adjusted mixed models showed that a point increase in OI was associated with a lower PFP score (β= -0.107, p= 0.006) and FI score (β= -0.009, p= 0.010). A point increase in OS was associated with a lower PFP score (β= -0.058, p= 0.016), but not FI score (β= -0.004, p= 0.064. These finding reveal distinct associations between peripheral and central measures of olfaction and frailty, highlighting the potential use of olfaction assessment as a biomarker and risk factor for frailty.

